# Epigallocatechin-3-gallate Attenuates Renal Damage by Suppressing Oxidative Stress in Diabetic db/db Mice

**DOI:** 10.1155/2016/2968462

**Published:** 2016-09-06

**Authors:** Xiu Hong Yang, Yu Pan, Xiao Li Zhan, Bao Long Zhang, Li Li Guo, Hui Min Jin

**Affiliations:** ^1^Division of Nephrology, Shanghai Pudong Hospital, Fudan University Pudong Medical Center, 2800 Gong Wei Road, Shanghai, China; ^2^Division of Nephrology, Shanghai No. 9 People's Hospital, Shanghai Jiao Tong University School of Medicine, Shanghai, China; ^3^The Institutes of Biomedical Sciences (IBS), Fudan University, Shanghai, China; ^4^Hemodialysis Center, Bao Shan Branch of No. 1 People's Hospital, Shanghai Jiao Tong University, Shanghai, China

## Abstract

Epigallocatechin-3-gallate (EGCG), extracted from green tea, has been shown to have antioxidative activity. In the present study, we evaluated the effect of EGCG on the kidney function in db/db mice and also tried to investigate the underlying mechanism of the renoprotective effects of EGCG in both animals and cells. EGCG treatment could decrease the level of urinary protein, 8-iso-PGF2a, and Ang II. Moreover, EGCG could also change the level of several parameters associated with oxidative stress. In addition, the protein expression levels of AT-1R, p22-phox, p47-phox, p-ERK1/2, p-p38 MAPK, TGF-*β*1, and *α*-SMA in diabetic db/db mice were upregulated, and all of these symptoms were downregulated with the treatment of EGCG at 50 and 100 mg/kg/d. Furthermore, the pathological changes were ameliorated in db/db mice after EGCG treatment. HK-2 cell-based experiments indicated that EGCG could inhibit the expression of MAPK pathways, which is the downstream effector of Ang II mediated oxidative stress. All these results indicated that EGCG treatment could ameliorate changes of renal pathology and delay the progression of DKD by suppressing hyperglycemia-induced oxidative stress in diabetic db/db mice.

## 1. Introduction

Based on the survey of International Diabetes Federation (IDF), the number of people with diabetes mellitus (DM) was as high as 415.0 million in 2015 all over the world [1]. In China, there are more than 98.4 million patients with diabetes in 2013, ranking first in the world, and the number is estimated to reach 143.0 million in 2035 [1]. Diabetes, a worldwide health problem, is defined as a group of metabolic diseases characterized by hyperglycemia. Diabetes is always associated with long-term damage, dysfunction, and failure of different organs, leading to the development of several complications [2, 3]. Diabetic kidney disease (DKD) is a kind of chronic microvascular complications associated with diabetes, and 40% or more diabetic patients have developed DKD despite current treatments [2, 3]. DKD is also a major cause of chronic renal failure [2, 3]. Therefore, it is of great importance to find new approaches to delay the progression of DKD so as to reduce the number of dialysis patients.

Several recent studies have suggested that oxidative stress is involved in the procession and development of kidney injury, including DKD [2, 4–6]. Oxidative stress refers to the increase of reactive oxygen species (ROS) production and/or the decrease of antioxidant production disordering the balance between oxidation and antioxidation, leading to renal injury [2, 6]. Through multiple mechanisms, ROS can cause kidney damage, such as inducing the expression of angiotensin II (Ang II), increasing the production of transforming growth factor-*β*1 (TGF-*β*1) and smooth muscle actin-*α* (*α*-SMA), and activating the mitogen-activated protein kinase (MAPK) cascade [7–12]. In addition, it has been demonstrated that the major source for the generation of ROS is nicotinamide adenine dinucleotide phosphate (NADPH) oxidase in diabetic conditions [5, 6, 13–15]. As reported previously, NADPH oxidase inhibitor can effectively restrain ROS generation, reduce the level of urinary protein, ameliorate the pathological changes of kidney, and delay the progression of DKD in the type 2 diabetic rat model [13].

Drinking tea is a common habit for Chinese people, which has a long history in China. Epigallocatechin-3-gallate (EGCG), a kind of green tea extract, is the major polyphenolic constituent present in green tea. Since it has been suggested to have anti-inflammatory, antioxidative, and hypoglycemic effects, EGCG has become a research hotpot in recent years [16–19]. But EGCG has been shown to exert prooxidative activities in some other studies [20, 21]. To date, a variety of studies have demonstrated that EGCG has salutary effects, but the precise mechanisms of EGCG are still unclear in DKD.

Therefore, we performed the present study to evaluate the effect of EGCG on the kidney function in db/db mice. Additionally, we also tried to investigate the underlying mechanism of the renoprotective effects of EGCG in both animals and cells.

## 2. Materials and Methods

### 2.1. Animals and Experimental Groups

Eight-week-old C57BLKS/J db/db mice (type 2 diabetic mice model) and their normal mice were purchased from Model Animal Research Center of Nanjing University (Nanjing, Jiangsu, China). The average weight of db/db mice was 33.7 ± 1.5 g. All the animal experimental procedures were approved by the Animal Care Committee of Fudan University. Mice were housed in plastic cages with a controlled temperature of 23–26°C, humidity of 50–55%, and a 12 h light/dark cycle. All the mice had free access to food and distilled water.

EGCG (>90% purity) was obtained from Sigma-Aldrich (St. Louis, MO, USA). Then Mice were allocated into 4 groups with the following treatment (*n* = 16, each): (1) normal, C57BLKS/J normal nontreated mice (gavage administration of 0.9% saline); (2) control, C57BLKS/J db/db nontreated mice (gavage administration of 0.9% saline); (3) EGCG A, C57BLKS/J db/db treated mice (gavage administration of EGCG 50 mg/kg/d); and (4) EGCG B, C57BLKS/J db/db treated mice (gavage administration of EGCG 100 mg/kg/d). The doses of EGCG were based on the previous researches [22]. Mice were sacrificed at week 4 and at week 8, with 8 animals killed each time in each group, respectively.

### 2.2. Oral Glucose Tolerance Test (OGTT)

After fasting for 16 h, a basal blood sample was collected from the tip of the tail of mice (*t* = 0 min). Then mice from all groups were subjected to an OGTT. Briefly, animals were given glucose (1 g/kg) by gavage and blood samples were collected from the tail vein of mice at 0, 15, 30, 60, 90, and 120 minutes after administration for the measurement of glucose. Fasting blood glucose concentration was measured with the OneTouch Basic glucose meter (LifeScan Canada Ltd., Burnaby, BC, Canada) and fasting plasma insulin was measured with mouse insulin ELISA kits (Crystal Chem, Downers Grove, IL, USA).

### 2.3. Measurement of Urine Protein, 8-Iso-prostaglandin F2*α* (8-Iso-PGF2a), and Ang II in the Renal Homogenate

Mice, one per metabolic cage, were placed for 24 h urine collection. Urine samples from all mice were centrifuged at 12000 rpm for 5 minutes. Then, the clear supernatant from urine samples was collected and stored at −80°C for further analysis. The 24-hour urinary protein was determined by Coomassie Blue Plus Protein Assay Kit (Pierce, Rockford, IL, USA). Moreover, renal Ang II concentration was measured with Mouse Angiotensin II Elisa Kit, which was obtained from USCN Life Science, Inc. (Wuhan, Hubei, China). Furthermore, 8-iso-PGF2a concentration in urine was measured by a commercial ELISA kit (Cayman, Ann Arbor, MI, USA).

### 2.4. Measurement of Reactive Oxygen Species (ROS), 8-Hydroxy-2′-deoxyguanosine (8-OHdG), Superoxide Dismutase (SOD), Malondialdehyde (MDA), Catalase (CAT), and 3-Nitrotyrosine Concentration in Kidney Homogenates

After animals were killed, the kidney was excised immediately and placed in ice-cold RIPA buffer (CST, Beverly, MA, USA) for homogenization using a tissue homogenizer. After centrifugation, supernatant was collected and used for analysis of generation of following parameters in kidney homogenates: ROS level was detected by a fluorometric assay using the 2′7′-dichlorodihydrofluorescein diacetate (DCFH-DA, Sigma-Aldrich, St. Louis, MO, USA) as a fluorescence probe; the levels of SOD, MDA, CAT, and 8-OHdG in kidney were measured with commercial kits (Nanjing Jiancheng, Nanjing, Jiangsu, China); and 3-nitrotyrosine, a marker for oxidative stress in the kidney, was detected by ELISA using a commercial kit (Millipore, Bedford, MA, USA).

### 2.5. Cell Cultures and Treatments

HK-2 cells, a line of human renal proximal tubular epithelial cells obtained from Bioresource Collection and Research Center (BCRC), were maintained in Dulbecco's modified Eagle's medium (DMEM; Invitrogen, Carlsbad, CA, USA) supplemented with 10% fetal bovine serum (FBS, HyClone, South Logan, UT, USA) and 2% antibiotics (HyClone, South Logan, UT, USA). The medium was changed every three days. When HK-2 cells grew to 80% confluence, they were cultured in serum-free medium for 24 h.

To investigate the underlying mechanism of renoprotective effects of EGCG, cells were randomly divided into 4 groups: (1) untreated group, (2) Ang II group (cells were treated with 1 *μ*M Ang II), (3) Ang II + EGCG A group (cells were first treated with 15 *μ*M EGCG for 6 h, followed by 1 *μ*M Ang II for 24 h), and (4) Ang II + EGCG B group (cells were first treated with 30 *μ*M EGCG for 6 h, followed by 1 *μ*M Ang II for 24 h). In addition, we also explored the role of PD98059 (ERK1/2 inhibitor, Sigma-Aldrich, St Louis, MO, USA) and SB202190 (p38 MAPK inhibitor, Sigma-Aldrich, St Louis, MO, USA) in Ang II mediated renal fibrosis; cells were then divided into five groups: (1) untreated group, (2) Ang II group (cells were treated with 1 *μ*M Ang II), (3) Ang II + PD98059 group (cells were first treated with 10 mM PD98059 for 6 h, followed by 1 *μ*M Ang II for 24 h), (4) Ang II + SB202190 group (cells were first treated with 10 mM SB202190 for 6 h, followed by 1 *μ*M Ang II for 24 h), and (5) Ang II + both groups (cells were first treated with 10 mM SB202190 and 10 mM PD98059 for 6 h, followed by 1 *μ*M Ang II for 24 h Ang II). The doses of Ang II, EGCG, PD98059, and SB202190 were based on the previous researches [23, 24].

### 2.6. Western Blot Analysis

All protein samples of both kidney tissue and cultured HK-2 cells were centrifuged at 14000 rpm for 10 minutes, and the clear supernatants were collected. Total protein concentrations in the supernatants were determined by the BCA method. Then, the protein was boiled at 98°C for 5 to 10 minutes and stored at −80°C for later analysis.

Total of 45 *μ*g protein (per sample) was electrophoresed via 10 or 12% SDS-PAGE and transferred onto nitrocellulose membranes (Bio-Rad, CA, USA). The membranes were then blocked with 5% nonfat milk or 5% BAS (bovine serum albumin) power and blocking buffer (1x Tris buffered saline and 0.1% Tween 20, Ph 7.4) at room temperature for 1 hour and next incubated with rabbit-anti-mouse primary antibodies overnight at 4°C: Ang II type 1 receptor (AT-1R, 1 : 1000), NADPH oxidase 1 (NOX-1, 1 : 1000), NOX-4 (1 : 1000), p22-phox (1 : 1000), p47-phox (1 : 1000), extracellular regulated protein kinases 1/2 (ERK1/2, 1 : 1000), p-ERK1/2 (1 : 1000), p38 MAPK (1 : 1000), p-p38 MAPK (1 : 500), TGF-*β*1 (1 : 100), *α*-SMA (1 : 100), and GAPDH (1 : 1000). The next day, after washing with Tris buffered saline for three times for 30 minutes (changed every 10 minutes), the membranes were incubated for 1 hour at room temperature with secondary antibodies (horseradish peroxidase-conjugated anti-rabbit IgG, 1 : 2000). For Western blot, the primary antibodies against AT-1R, p22-phox, p47-phox, TGF-*β*1, *α*-SMA, and GAPDH were purchased from Santa Cruz Biotechnology (Santa Cruz, CA, USA); those against p-ERK1/2, ERK1/2, p-p38 MAPK, p38 MAPK, and secondary antibodies were obtained from Cell Signaling Technology, Inc. (Beverly, MA, USA); antibodies against NOX1 and NOX4 were obtained from Abcam Company (Cambridge, MA, USA). Finally, the protein bands were visualized using SuperSignal West Femto Substrate (Pierce, Rockford, IL, USA).

### 2.7. Histopathology and Immunohistochemistry of Kidney

Histopathology and immunohistochemistry of kidney were performed as described previously [25]. The kidney tissue sections were kept in 4% paraformaldehyde solution and paraffin-embedded and then were cut into 3 *μ*M thickness. The renal pathological changes were examined by Periodic Acid-Schiff (PAS) staining. The expressions of TGF-*β*1 (1 : 100) and *α*-SMA (1 : 100) were evaluated by immunohistochemistry. The percentage of positive staining area was quantified using Image-Pro Plus 6.0 software (Media Cybernetics, Silver Spring, MD, USA).

### 2.8. Statistical Analysis

The Stata 10.0 statistical software (Stata) was used for all statistical analysis. Results were expressed as means ± SEM. Differences among groups were subjected to a one-way analysis of variance (ANOVA) and *P* < 0.05 was considered significantly different.

## 3. Results

### 3.1. Detection of Body Weight (BW), Kidney Weight/Body Weight (KW/BW), Fasting Plasma Glucose, and Insulin Levels in Mice from Different Groups

Table 1 shows the changes in BW, KW/BW, the level of glucose, and insulin level in plasma of mice after administration by gavage with or without EGCG. At baseline, the BW of db/db mice was higher than normal mice. At four and eight weeks after oral administration of EGCG, the BW of db/db mice was still higher than normal mice, and there was no significant difference in db/db mice of each group. However, db/db mice treated with EGCG at 50 and 100 mg/kg/d had a significantly lower KW/BW when compared to nontreated db/db mice (*P* < 0.01). Compared with the normal group, the blood glucose level of db/db mice was obviously higher, which persistently increased during the whole study, whereas the level of fasting plasma glucose was obviously decreased (*P* < 0.01) and the level of fasting plasma insulin was significantly increased (*P* < 0.01) in db/db mice treated with EGCG compared to nontreated db/db mice (*P* < 0.01) after the treatment for 4 and 8 weeks.

**Figure 1:**
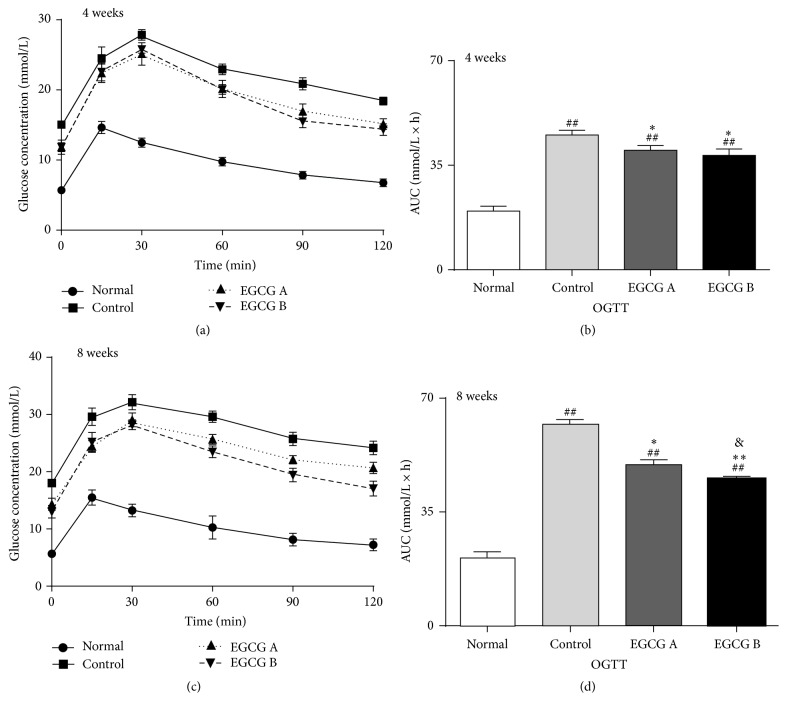
Glucose metabolic profile in db/db mice versus control mice. Diabetic db/db mice were treated with or without EGCG for 4 or 8 weeks, and OGTT (glucose, 1 g/kg) were performed. Glucose concentrations (a and c) and the AUC of OGTT (b and d) were shown. Normal: nontreated C57BL mice; control: C57BLKS/J db/db nontreated mice; EGCG A, db/db mice treated with EGCG of 50 mg/kg/d; EGCG B, db/db mice treated with EGCG of 100 mg/kg/d; values are means ± SEM. ^##^ *P* < 0.01 versus normal; ^*∗*^ *P* < 0.05 and ^*∗∗*^ *P* < 0.01 versus control; ^&^ *P* < 0.05 versus EGCG A. At week 4, *n* = 16 in each group; at week 8, *n* = 8 in each group.

In addition, the area under the curve (AUC) for OGTT in db/db mice was also higher than normal group, yet the AUC was significantly lower in db/db mice with the treatment of EGCG than nontreated db/db mice (Figures 1(a), 1(b), 1(c), and 1(d), *P* < 0.05). Furthermore, the AUC of db/db mice treated with EGCG at 100 mg/kg/d was lower in comparison with the group of db/db mice treated with EGCG at 50 mg/kg/d after the oral administration for 8 weeks (Figures 1(c) and 1(d), *P* < 0.05).

### 3.2. Changes in Urine Protein, 8-Iso-PGF2a, and Renal Homogenate Ang II after Different Treatment

Urine collection was performed 24 h after treatment in mice and levels of urine protein, 8-iso-PGF2a in urine, and Ang II of kidney were summarized in Table 2. The level of 24 h urine protein was reduced remarkably in db/db mice treated with EGCG compared to nontreated db/db mice during 8-week period (*P* < 0.05). Equally, EGCG treatment significantly decreased the level of 8-iso-PGF2a in urine compared to the nontreated db/db mice (*P* < 0.05). The significant decrease in Ang II level in kidney was also observed in db/db mice treated with EGCG compared to nontreated db/db mice (*P* < 0.05).

### 3.3. Changes in ROS, 8-OHdG, SOD, MDA, CAT, and 3-Nitrotyrosine in Kidney Homogenates in Mice after Different Treatment

We also explored the changes of several parameters in kidney homogenates in mice from different groups and the results were displayed in Table 3. Compared to the normal mice, there were significant changes (significant increase or decrease) in ROS, 8-OHdG, SOD, MDA, CAT, and 3-nitrotyrosine levels in db/db mice (*P* < 0.05). However, EGCG treatment (both concentrations of 50 mg/kg/d and 100 mg/kg/d) could obviously ameliorate the changes in the level of above parameters in db/db mice (*P* < 0.05) during the 8-week study period.

### 3.4. Changes in Expression of AT-1R, TGF-*β*1, p22-phox, and p47-phox in Kidney Tissue in Mice after Different Treatment

**Figure 2:**
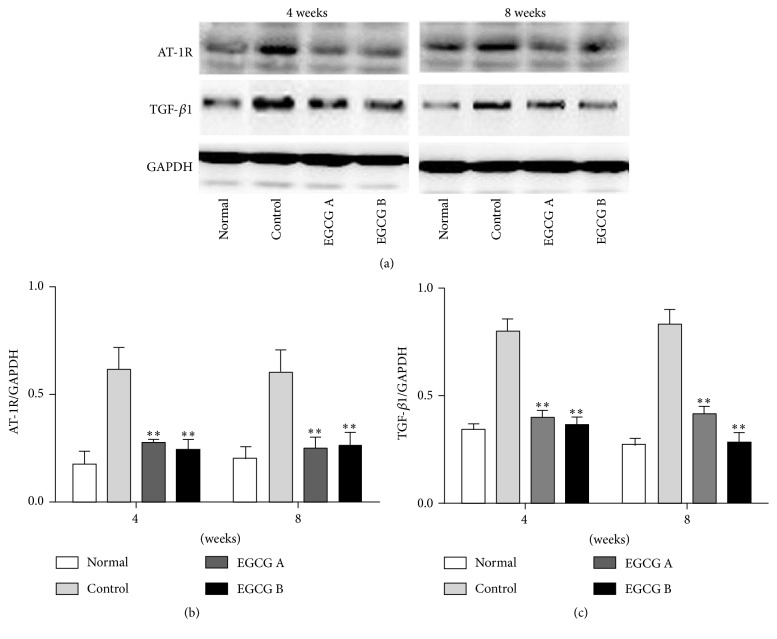
The protein expressions of AT-1R (b) and TGF-*β*1 (c) in renal tissue of mice detected by Western blot. Normal: nontreated C57BL mice; control: C57BLKS/J db/db nontreated mice; EGCG A, db/db mice treated with EGCG of 50 mg/kg/d; EGCG B, db/db mice treated with EGCG of 100 mg/kg/d; values are means ± SEM. ^*∗∗*^ *P* < 0.01 versus control. At both week 4 and week 8, *n* = 8 in each group.

As shown in Table 2, the renal Ang II level was increased in mice after treatment with EGCG. Moreover, we also explored the effects of EGCG on AT-1R, which is the receptor of Ang II. Following our results, the level of AT-1R was significantly downregulated in mice after oral administration of EGCG for 4 and 8 weeks (Figures 2(a) and 2(c), *P* < 0.01). Therefore, EGCG may play a role similar to Ang II type 1 receptor blocker. Likewise, the protein expression level of TGF-*β*1 in renal tissue was also obviously downregulated after treatment of EGCG (Figures 2(a) and 2(b), *P* < 0.01).

**Figure 3:**
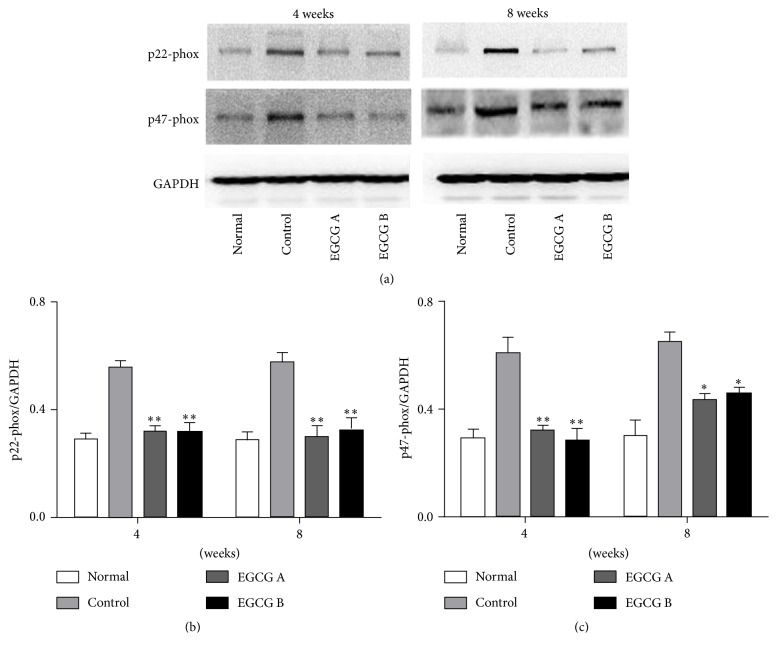
The protein expressions of p22-phox (b) and p47-phox (c) in renal tissue of mice detected by Western blot. Normal: nontreated C57BL mice; control: C57BLKS/J db/db nontreated mice; EGCG A, db/db mice treated with EGCG of 50 mg/kg/d; EGCG B, db/db mice treated with EGCG of 100 mg/kg/d; values are means ± SEM. ^*∗*^ *P* < 0.05 and ^*∗∗*^ *P* < 0.01 versus control. At both week 4 and week 8, *n* = 8 in each group.

Hyperglycemia-induced oxidative stress plays an important role in the procession and development of DKD, which was confirmed by Western blot analysis in our study. Compared with the normal group, p22-phox and p47-phox (both were NADPH oxidase subunits) were upregulated in nontreated db/db mice, while EGCG treatment significantly reduced the protein expression of p22-phox and p47-phox in db/db mice (Figures 3(a), 3(b), and 3(c), *P* < 0.05).

### 3.5. Changes in the Expression of MAPK Signaling Pathway

**Figure 4:**
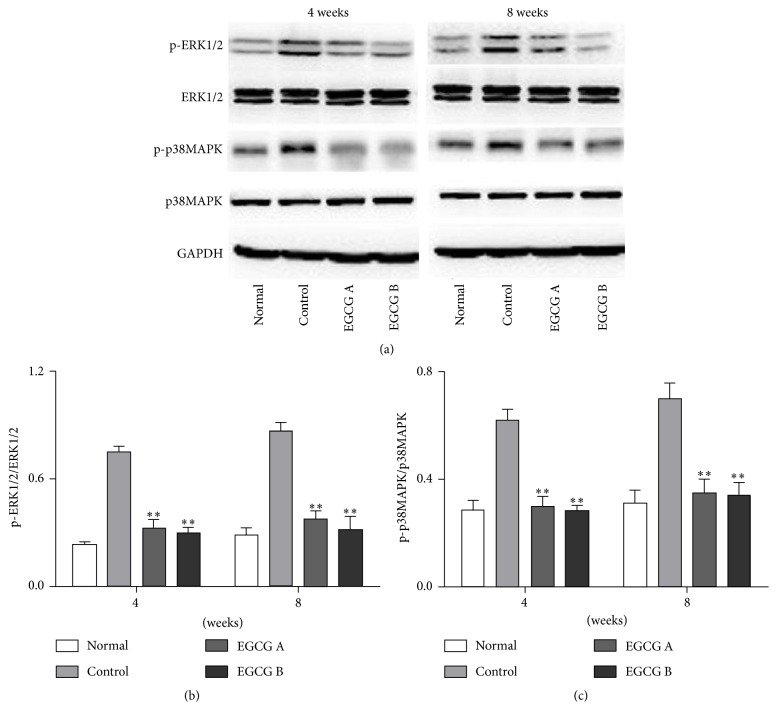
The protein expression of p-ERK1/2 (b) and p-P38 MAPK (c) in renal tissue of mice detected by Western blot. Normal: nontreated C57BL mice; control: C57BLKS/J db/db nontreated mice; EGCG A, db/db mice treated with EGCG of 50 mg/kg/d; EGCG B, db/db mice treated with EGCG of 100 mg/kg/d; values are means ± SEM. ^*∗∗*^ *P* < 0.01 versus control. At both week 4 and week 8, *n* = 8 in each group.

Given that the oxidative stress may activate the MAPK cascade, we explored the changes in expression of p-ERK1/2 and p-p38 MAPK (both are important members of MAPK family) in kidney tissue in mice after EGCG treatment (Figure 4(a)). In this study, we found that the protein expressions of p-ERK1/2 and p-p38 MAPK were obviously increased in nontreated db/db mice compared to those in the kidney tissue in normal mice, and both of them were significantly reduced in mice after treatment of EGCG (Figures 4(b) and 4(c), *P* < 0.05).

### 3.6. EGCG Ameliorated Renal Damage and Fibrosis in db/db Mice

**Figure 5:**
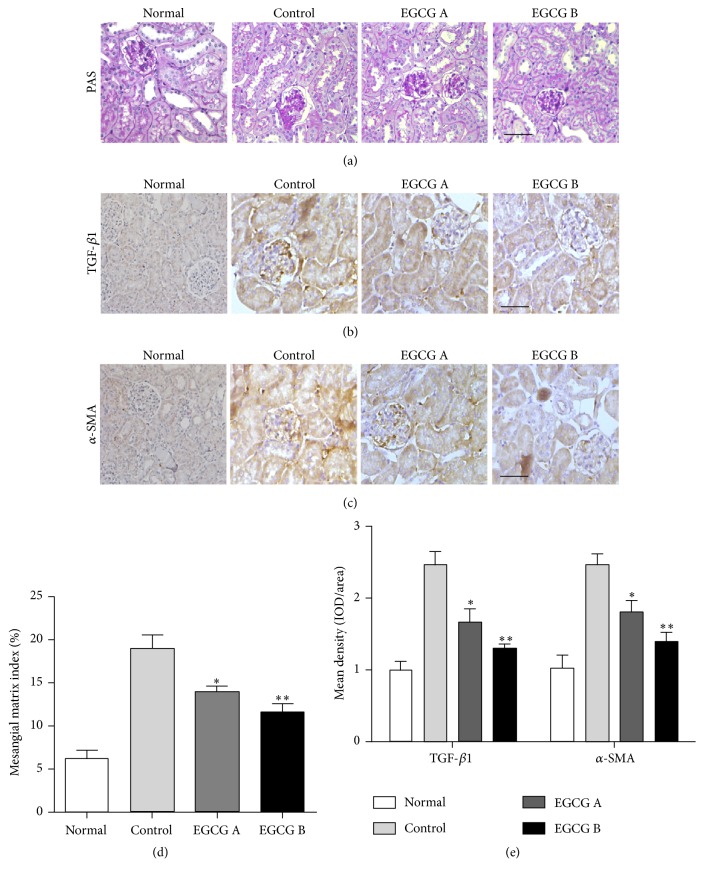
Effect of EGCG on renal pathological changes and fibrosis. Renal pathological changes were examined by PAS staining (a and d). The protein expression levels of TGF-*β*1 and *α*-SMA were evaluated by immunohistochemistry (b, c, and e). Scale bar: 50 *μ*m; *n* = 8 in each group. Normal: nontreated C57BL mice; control: C57BLKS/J db/db nontreated mice; EGCG A, db/db mice treated with EGCG of 50 mg/kg/d; EGCG B, db/db mice treated with EGCG of 100 mg/kg/d; values are means ± SEM. ^*∗*^ *P* < 0.05 and ^*∗∗*^ *P* < 0.01 versus control.

The renoprotective effects of EGCG in ameliorating renal damage and fibrosis were investigated by PAS and immunohistochemical staining (Figures 5(a), 5(b), and 5(c)). Compared to the normal mice, there was an increase of glomerular volume and mesangial matrix expansion, and EGCG treatment could attenuate these histological changes in db/db mice confirmed by PAS staining (Figure 5(a)). Results from statistical analysis indicated that EGCG could significantly reduce mesangial matrix index in db/db mice (Figure 5(d), *P* < 0.05). Additionally, the expression levels of TGF-*β*1 and *α*-SMA, which were associated with the development of fibrosis, were significantly downregulated after EGCG treatment in db/db mice compared to those in nontreated mice (Figures 5(b), 5(c), and 5(e), *P* < 0.05).

### 3.7. Effects of EGCG on Ang II Induced Renal Kidney Injury in HK-2 Cells

**Figure 6:**
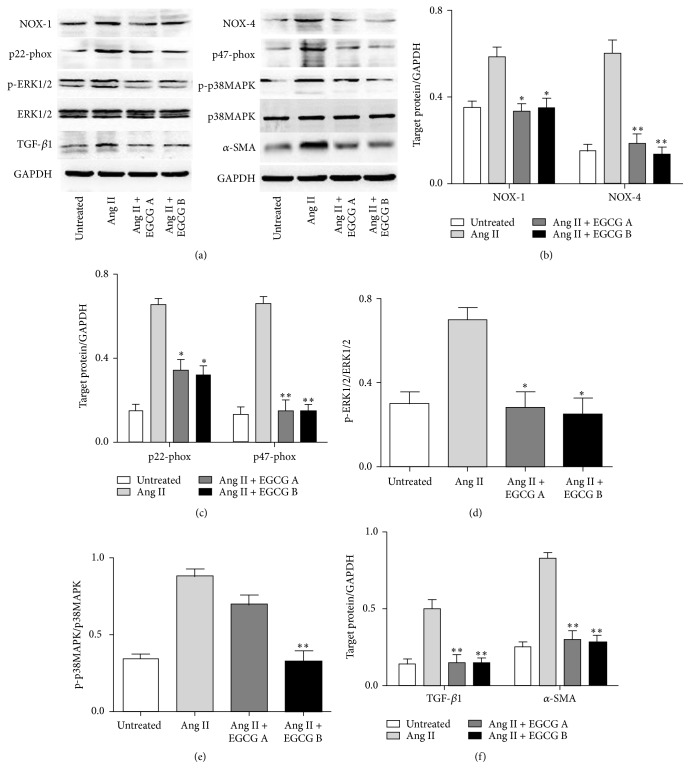
The protein expression of NOX-1 and NOX-4 (b), p22-phox and p47-phox (c), p-ERK1/2 (d), p-P38 MAPK (e), and TGF-*β*1 and *α*-SMA (f) of HK-2 cell after exposure to Ang II and EGCG detected by Western blot analyses. HK-2 cells were pretreated with 15 or 30 *μ*M of EGCG for 6 h and then stimulated with Ang II (1 *μ*M) for 24 hours. Values are means ± SEM. ^*∗*^ *P* < 0.05 and ^*∗∗*^ *P* < 0.01 versus control.

**Figure 7:**
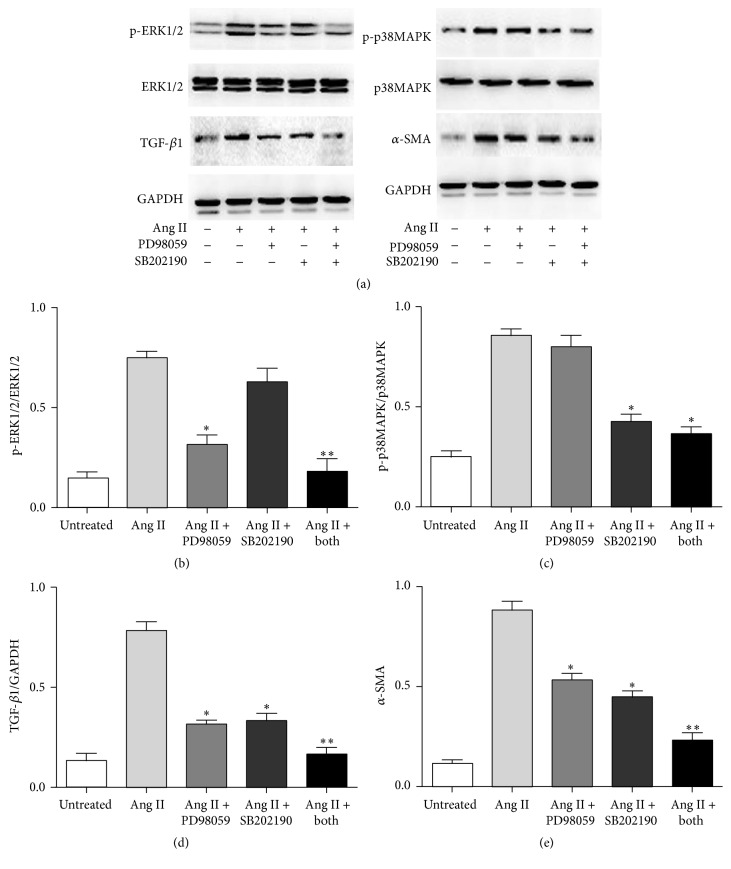
The protein expression of p-ERK1/2 (b), p-P38 MAPK (c), TGF-*β*1 (d), and *α*-SMA (e) of HK-2 cell after exposure to Ang II, PD98059, and SB202190. HK-2 cells were pretreated with PD98059 (ERK1/2 inhibitor) and/or SB202190 (p38 MAPK inhibitor) (all 10 mM) for 6 h and then stimulated with Ang II (1 *μ*M) for 24 hours. Values are means ± SEM. ^*∗*^ *P* < 0.05 and ^*∗∗*^ *P* < 0.01 versus control.

To explore the underlying mechanism of EGCG in renoprotection, we investigated the role of EGCG in Ang II mediated renal injury pathway in HK-2 cells. Following our results in Figure 6, after intervention with EGCG, the increased expression of NOX-1 (Figures 6(a) and 6(b)), NOX-4 (Figures 6(a) and 6(b)), p22-phox (Figures 6(a) and 6(c)), p47-phox (Figures 6(a) and 6(c)), p-ERK1/2 (Figures 6(a) and 6(d)), p-P38 MAPK (Figures 6(a) and 6(e)), TGF-*β*1 (Figures 6(a) and 6(f)), and *α*-SMA (Figures 6(a) and 6(f)) induced by Ang II was significantly downregulated (*P* < 0.05). We also explored the role of PD98059 and SB202190 in Ang II mediated renal fibrosis. Based on our results, PD98059 and SB202190 can significantly decrease the expression of p-ERK1/2 (Figures 7(a) and 7(b)), p-P38 MAPK (Figures 7(a) and 7(c)), TGF-*β*1 (Figures 7(a) and 7(d)), and *α*-SMA (Figures 7(a) and 7(e)) even in the presence of Ang II (*P* < 0.05). Hence, EGCG is supposed to play a role in Ang II induced renal injury similar to PD98059 and SB202190.

## 4. Discussion 

In this study, we founded that EGCG treatment at 50 or 100 mg/kg/d for 4 and 8 weeks could decrease the level of plasma glucose and also increase the level of plasma insulin in db/db mice. Moreover, 24 h urinary protein, 8-iso-PGF2a, and renal Ang II levels were also reduced by EGCG treatment. EGCG could also change the level of several parameters in renal homogenate including ROS, 8-OHdG, SOD, MDA, CAT, and 3-nitrotyrosine in db/db mice. In addition, EGCG treatment at dose of 50 and 100 mg/kg/d could also downregulate the expression of several factors involved in the signaling pathways activated by ROS production, including AT-1R, p22-phox, p47-phox, p-ERK1/2, p-p38 MAPK, TGF-*β*1, and *α*-SMA. Meanwhile, the pathological changes in diabetic db/db mice such as renal injury and fibrosis could be also ameliorated after EGCG treatment. Thus, we supposed that EGCG, a constituent of green tea, had beneficial effects on the kidney of diabetic db/db mice.

To date, numerous studies have suggested that oxidative stress can lead to kidney injury and accelerate the procession and development of DKD [2, 4–6]. Oxidative stress of DKD is mainly derived from hyperglycemia. Hence, controlling hyperglycemia is supposed to be an effective approach to slow the procession and development of DKD. Previous studies have shown that EGCG has antihyperglycemia effect [16, 19, 26]. However, results from our study demonstrated that EGCG plays a role in regulating glycol metabolism. The level of fasting plasma glucose was decreased and the level of fasting plasma insulin was increased in db/db mice after treatment of EGCG. Furthermore, the AUC was significantly lower in db/db mice treated with EGCG compared to nontreated db/db mice. Interestingly, all these beneficial effects of EGCG were not obviously dose-dependent. On the other hand, there was no significant difference between the doses of 50 mg/kg/d and 100 mg/kg/d.

ROS production is a major process of oxidative stress, which has been shown to participate in DKD through various mechanisms, such as stimulating the expression of Ang II, inducing the production of TGF-*β*1 and *α*-SMA, and activating the MAPK cascade [7–11].

DKD is once considered to be caused by combined effects of hemodynamic changes and metabolic factors. This theory cannot clearly elucidate the pathogenesis of DKD, while hemodynamic factors are supposed to play an important role in the development of DKD [27, 28] learning from it. As described previously, the hemodynamic changes in kidney are related to the progression of DKD, and ROS takes part in these changes of renal hemodynamics in diabetic conditions, which stimulates the expression of renin angiotensin system (RAS) [27, 29, 30]. ROS-induced activation of Ang II, a crucial component of RAS, is able to increase intraglomerular pressure and activate the intracellular second messengers, which eventually results in the upregulation of urinary protein and renal fibrosis [31]. In addition, the increased level of Ang II accelerates the production of ROS [7]. AT-1R, the important receptor of Ang II, has been shown to play a significant role in increasing the urinary protein level [32]. It has been reported that, in diabetic conditions, there is a vicious circle: ROS-angiotensinogen-Ang II-AT-1R-ROS [33]. Moreover, studies have confirmed that AT-1R blocker remarkably alleviated the level of urinary protein of DKD patients [33]. Based on the results from our study, EGCG not only obviously inhibited the level of ROS-induced Ang II expression in the renal homogenate but also significantly suppressed the protein expression of AT-1R. The effect of EGCG on AT-1R has been reported in only two studies. One study indicated that EGCG significantly reduced the expression of AT-1R mRNA in the liver of SHRSP-ZF rats [34], and the other demonstrated that EGCG could restore AT-1R mRNA expression to normal level which was decreased by Ang II stimulation in rat cardiac fibroblasts. In this study, it was the first time to show that EGCG decreased the protein expression of AT-1R in renal tissues of DKD [35]. Hence, EGCG may function as an AT-1R blocker. Further study should be performed to compare the effects of EGCG with olmesartan (AT-1R specific blocker) on kidney pathological changes in db/db mice.

Oxidative stress is considered the major culprit in kidney tissues. NADPH oxidase is abundantly distributed in mesangial cells, and renal tubular cells are also the targeted cells of ROS. ROS can induce the apoptosis of both mesangial cells and tubular cells in kidney, leading to pathological changes, eventually causing glomerular sclerosis and tubule-interstitial fibrosis. Studies have demonstrated that a major source for generation of ROS is NADPH oxidase in high-glucose conditions [5, 6, 13–15]. It has been reported that NADPH oxidase inhibitor can effectively inhibit the generation of ROS and delay the progression of DKD in the type 2 diabetic rat model [13]. In this study, our data demonstrated that the protein expression levels of p22-phox and p47-phox were significantly decreased in db/db mice after EGCG treatment. Similarly, the pathological changes in kidney were alleviated in mice after the oral administration with EGCG. Furthermore, the levels of 24 h urinary protein and 8-iso-PGF2a and renal Ang II were also reduced with the treatment of EGCG. Considering these results, EGCG exerts antioxidant effect in DKD of db/db mice. However, some other studies show that EGCG has the ability to exert prooxidative activities [20, 21]. These discrepancies may be caused by the different mechanism of EGCG involved in different diseases. It is well known that molecules play different roles, even totally opposite roles, in different cells or tissues. EGCG may have prooxidative activities in other diseases, while it shows an antioxidative effect in renal injury.

Oxidative stress-induced inflammatory response is a major pathomechanism of DKD. Moreover, the increase of inflammatory cytokine levels under high glucose conditions in return could induce a further increase of oxidative stress forming a vicious cycle [36]. It has been reported that oxidative stress is a major contributor to the increase of TGF-*β*1 and *α*-SMA in DKD through direct or indirect ways [9, 36]. Both TGF-*β*1 and *α*-SMA are hypertrophic and fibrogenic cytokines which play a major role in glomerular hypertrophy and mesangial matrix expansion and finally lead to end-stage renal disease [36]. In addition, NADPH oxidase inhibitor effectively decreases TGF-*β*1 and *α*-SMA levels [37]. Furthermore, MAPK signaling pathways can be activated by ROS and participate in regulation of the inflammatory cytokines and processes [11]. It has been reported that ERK1/2 and p38 MAPK, members of MAPK family, are crucial to mediate cellular responses such as inducing the proliferation, differentiation, and apoptosis of kidney cells and activating inflammatory processes [11, 33]. Therefore, TGF-*β*1, *α*-SMA, and MAPK signaling pathways which are stimulated by ROS are closely associated with the development of DKD. In this study, the db/db nontreated mice displayed obviously increased protein expression levels of p-ERK1/2 and p-p38 MAPK confirmed by Western blot and levels of TGF-*β*1 and *α*-SMA determined by immunohistochemistry compared to normal group. On the other hand, the increase levels of TGF-*β*1, *α*-SMA, p-ERK1/2, and p-p38 MAPK were inhibited by EGCG in diabetic conditions. Furthermore, in order to further explore the underlying molecular mechanism of renoprotective effects of EGCG, we performed a cell-based study in HK-2 cells. Based on our results, EGCG could downregulate the expression of several factors involved in Ang II mediated oxidative stress in kidney including NOX-1, NOX-4, p22-phox, p47-phox, p-ERK1/2, p-P38 MAPK, TGF-*β*1, and *α*-SMA. EGCG is supposed to play a role in Ang II induced renal injury similar to PD98059 and SB202190, both of which are blockers of downstream molecules of Ang II pathway.

**Figure 8:**
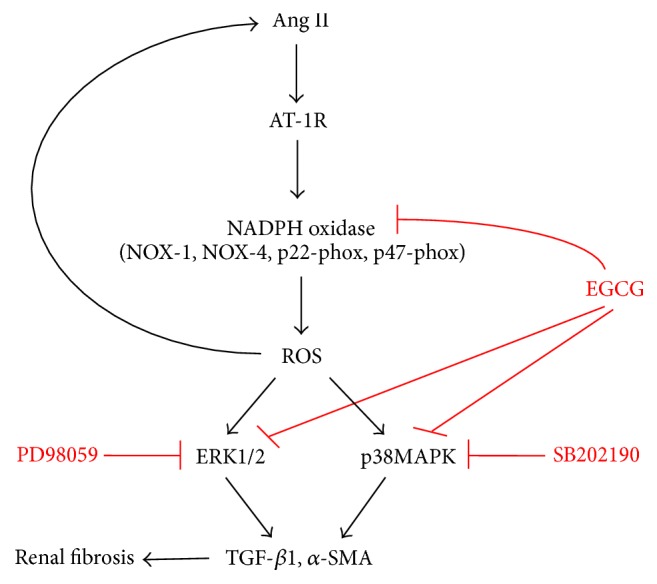
Diagram of the underlying mechanisms of EGCG disturbing Ang II induced oxidative stress and renal fibrosis.

Considering the effects of EGCG in Ang II mediated oxidative stress in DKD in our study, we plotted a diagram elucidating the possible function of EGCG in Ang II regulated pathways, as shown in Figure 8. The renoprotective effects of EGCG might be realized by two pathways: one is functioning as a NADPH oxidase inhibitor, directly suppressing the production of ROS, and the other is inhibiting the expression of downstream molecules of Ang II mediated pathway, directly downregulating the production of effectors for renal fibrosis. Other possible mechanisms of EGCG such as function as an AT-1R blocker should be explored in further studies.

In summary, our results suggested that EGCG has renoprotective effects in diabetic db/db mice through disturbing Ang II mediated oxidative stress. These effects of EGCG are realized by several mechanisms including directly suppressing the activity of NADPH oxidase and inhibiting MAPK cascade involved in Ang II pathway.

## Figures and Tables

**Table 1:** Changes in BW, KW/BW, glucose, and insulin levels in mice after different treatment.

	Normal	Control	EGCG A	EGCG B
BW (g)				
Baseline	25.2 ± 0.6	33.2 ± 0.8^##^	33.4 ± 1.0^##^	33.3 ± 1.1^##^
Week 4	31.4 ± 1.3	39.8 ± 1.4^##^	38.9 ± 1.9^##^	39.5 ± 1.7^##^
Week 8	35.3 ± 1.1	45.6 ± 1.2^##^	44.2 ± 1.8^##^	44.3 ± 3.1^##^
KW/BW (mg/g)				
Baseline	3.5 ± 0.4	6.5 ± 0.2^##^	6.3 ± 0.5^##^	6.4 ± 0.4^##^
Week 4	3.6 ± 0.4	8.1 ± 0.3^##^	7.1 ± 0.8^##**∗∗**^	7.3 ± 0.1^##**∗∗**^
Week 8	4.0 ± 0.6	8.4 ± 0.1^##^	7.4 ± 0.2^##**∗∗**^	7.5 ± 0.1^##**∗∗**^
Glucose (mmol/L)				
Baseline	4.9 ± 0.3	11.5 ± 0.9^##^	11.3 ± 1.1^##^	11.5 ± 1.1^##^
Week 4	4.5 ± 0.9	15.1 ± 0.5^##^	12.8 ± 1.0^##**∗∗**^	12.1 ± 0.6^##**∗∗**^
Week 8	4.7 ± 0.8	17.2 ± 0.8^##^	14.4 ± 1.0^##**∗∗**^	14.2 ± 0.7^##**∗∗**^
Insulin (ng/mL)				
Baseline	3.3 ± 1.3	5.6 ± 1.4^#^	5.4 ± 1.1^#^	5.7 ± 0.8^#^
Week 4	3.2 ± 0.7	5.5 ± 1.1^#^	7.8 ± 0.8^##*∗*^	8.0 ± 0.9^##**∗∗**^
Week 8	3.5 ± 1.1	5.2 ± 1.7^#^	11.2 ± 2.2^##**∗∗**^	12.4 ± 1.6^##**∗∗**^

Note: normal, C57BLKS/J normal nontreated mice; control, C57BLKS/J db/db nontreated mice; EGCG A, db/db mice treated with EGCG of 50 mg/kg/d; EGCG B, db/db mice treated with EGCG of 100 mg/kg/d; BW, body weight; KW/BW, kidney weight/body weight; glucose, fasting plasma glucose; insulin, fasting plasma insulin; ^#^ *P* < 0.05 and ^##^ *P* < 0.01 versus normal; ^*∗*^ *P* < 0.05 and ^*∗∗*^ *P* < 0.01 versus control; values are means ± SEM. At baseline and at week 4, *n* = 16 in each group; at week 8, *n* = 8 in each group.

**Table 2:** Changes of 24-hour urinary protein, urinary 8-iso-PGF2a, and renal Ang II levels in urine 24 h after different treatment.

	Normal	Control	EGCG A	EGCG B
24-hour urinary protein (mg)				
Baseline	1.2 ± 0.3	5.8 ± 0.6^##^	5.9 ± 0.9^##^	5.7 ± 1.1^##^
Week 4	1.1 ± 0.7	7.5 ± 0.2^##^	6.7 ± 0.1^##**∗∗**^	6.7 ± 0.5^##**∗∗**^
Week 8	1.3 ± 0.5	11.9 ± 1.3^##^	8.8 ± 1.0^##**∗**^	8.6 ± 1.1^##**∗**^
Urinary 8-iso-PGF2a (ng/d)				
Baseline	38.5 ± 5.3	84.6 ± 8.5^##^	87.2 ± 11.3^##^	86.8 ± 12.1^##^
Week 4	39.8 ± 3.6	150.4 ± 11.9^##^	107.3 ± 12.1^##**∗∗**^	110.6 ± 9.2^##**∗∗**^
Week 8	38.7 ± 4.4	176.8 ± 10.1^##^	138.5 ± 8.3^##**∗∗**^	126.7 ± 9.5^##**∗∗**^
Renal Ang II (ng/L)				
Baseline	177.6 ± 20.2	238.3 ± 14.2^##^	240.5 ± 19.8^##^	238.9 ± 16.6^##^
Week 4	182.4 ± 12.1	346.2 ± 22.7^##^	297.5 ± 15.5^##**∗**^	308.9 ± 7.3^##**∗**^
Week 8	191.3 ± 23.9	405.8 ± 7.2^##^	367.4 ± 5.4^##**∗∗**^	383.7 ± 9.6^##**∗**^

Note: normal, C57BLKS/J normal nontreated mice; control, C57BLKS/J db/db nontreated mice; EGCG A, db/db mice treated with EGCG of 50 mg/kg/d; EGCG B, db/db mice treated with EGCG of 100 mg/kg/d; urinary 8-iso-PGF2a, urinary 8-iso-prostaglandin F2*α*; Ang II: the renal angiotensin II concentration; ^##^ *P* < 0.01 versus normal; ^**∗**^ *P* < 0.05 and ^**∗∗**^ *P* < 0.01 versus control; values are means ± SEM. At baseline and at week 4, *n* = 16 in each group; at week 8, *n* = 8 in each group.

**Table 3:** Changes in ROS, 8-OHdG, SOD, MDA, CAT, and 3-nitrotyrosine in kidney homogenates in mice after different treatment.

	Normal	Control	EGCG A	EGCG B
ROS (U/mL)				
Baseline	201.4 ± 17.5	279.1 ± 17.8^##^	280.4 ± 20.7^##^	277.6 ± 23.2^##^
Week 4	203.9 ± 10.7	372.2 ± 20.2^##^	316.3 ± 18.1^##**∗∗**^	304.5 ± 23.5^##**∗∗**^
Week 8	218.4 ± 15.5	489.9 ± 20.3^##^	375.8 ± 21.4^##**∗∗**^	352.6 ± 19.1^##**∗∗**^
8-OHdG (pg/mg protein)				
Baseline	810.7 ± 32.6	958.3 ± 32.5^##^	975.2 ± 30.6^##^	969.2 ± 22.3^##^
Week 4	816.7 ± 35.4	1398.2 ± 33.7^##^	1179.5 ± 28.4^##**∗**^	1163.6 ± 34.5^##**∗∗**^
Week 8	823.1 ± 30.4	1757.8 ± 21.9^##^	1474.5 ± 25.3^##**∗∗**^	1365.3 ± 28.6^##**∗∗**^
SOD (U/mg protein)				
Baseline	60.8 ± 4.8	49.3 ± 7.1^#^	50.4 ± 5.5^#^	49.9 ± 6.2^#^
Week 4	61.3 ± 7.1	34.2 ± 7.2^##^	43.5 ± 3.5^#**∗**^	44.9 ± 4.3^#**∗**^
Week 8	59.1 ± 5.2	30.4 ± 4.7^##^	38.6 ± 6.4^#**∗∗**^	41.4 ± 3.6^#**∗∗**^
MDA (nM/mg protein)				
Baseline	14.6 ± 4.4	16.8 ± 3.3^#^	16.4 ± 3.7^#^	16.7 ± 5.6^#^
Week 4	15.3 ± 3.7	23.6 ± 3.5^#^	19.8 ± 1.6^#**∗**^	18.5 ± 4.3^#**∗∗**^
Week 8	15.7 ± 1.2	28.7 ± 4.1^#^	22.6 ± 2.9^#**∗**^	21.2 ± 5.3^#**∗**^
CAT (U/mg protein)				
Baseline	26.2 ± 2.2	23.0 ± 1.4^#^	23.3 ± 2.3^#^	23.1 ± 2.8^#^
Week 4	26.4 ± 1.2	15.9 ± 2.9^##^	21.5 ± 1.7^#**∗∗**^	21.3 ± 2.1^#**∗∗**^
Week 8	25.1 ± 1.8	13.6 ± 2.7^##^	17.4 ± 1.5^#**∗**^	18.3 ± 2.4^#**∗∗**^
3-nitrotyrosine (*μ*g/mL)				
Baseline	18.7 ± 2.8	20.4 ± 1.1^#^	20.3 ± 1.0^#^	21.1 ± 2.4^#^
Week 4	18.5 ± 2.6	29.5 ± 3.3^##^	24.8 ± 0.9^#**∗**^	22.3 ± 1.8^#**∗**^
Week 8	20.4 ± 3.1	36.7 ± 2.8^##^	30.2 ± 1.5^#**∗**^	31.4 ± 2.7^#**∗**^

Note: normal, C57BLKS/J normal nontreated mice; control, C57BLKS/J db/db nontreated mice; EGCG A, db/db mice treated with EGCG of 50 mg/kg/d; EGCG B, db/db mice treated with EGCG of 100 mg/kg/d; ROS, reactive oxygen species; 8-OHdG, 8-hydroxy-2′-deoxyguanosine; SOD, superoxide dismutase; CAT, catalase; MDA, malondialdehyde. ^#^ *P* < 0.05 and ^##^ *P* < 0.01 versus normal; ^**∗**^ *P* < 0.05 and ^**∗∗**^ *P* < 0.01 versus control; values are means ± SEM. At baseline and at week 4, *n* = 16 in each group; at week 8, *n* = 8 in each group.
